# Defective Resensitization in Human Airway Smooth Muscle Cells Evokes β-Adrenergic Receptor Dysfunction in Severe Asthma

**DOI:** 10.1371/journal.pone.0125803

**Published:** 2015-05-29

**Authors:** Manveen K. Gupta, Kewal Asosingh, Mark Aronica, Suzy Comhair, Gaoyuan Cao, Serpil Erzurum, Reynold A. Panettieri, Sathyamangla V. Naga Prasad

**Affiliations:** 1 Department of Molecular Cardiology, Lerner Research Institute, Cleveland Clinic, Cleveland, Ohio, United States of America; 2 Department of Pathology, Lerner Research Institute, Cleveland Clinic, Cleveland, Ohio, United States of America; 3 Airways Biology Initiative, Pulmonary, Allergy and Critical Care Division, University of Pennsylvania, Philadelphia, Pennsylvania, United States of America; Texas A& M University Health Science Center, UNITED STATES

## Abstract

β_2_-adrenergic receptor (β_2_AR) agonists (β_2_-agonist) are the most commonly used therapy for acute relief in asthma, but chronic use of these bronchodilators paradoxically exacerbates airway hyper-responsiveness. Activation of βARs by β-agonist leads to desensitization (inactivation) by phosphorylation through G-protein coupled receptor kinases (GRKs) which mediate β-arrestin binding and βAR internalization. Resensitization occurs by dephosphorylation of the endosomal βARs which recycle back to the plasma membrane as agonist-ready receptors. To determine whether the loss in β-agonist response in asthma is due to altered βAR desensitization and/or resensitization, we used primary human airway smooth muscle cells (HASMCs) isolated from the lungs of non-asthmatic and fatal-asthmatic subjects. Asthmatic HASMCs have diminished adenylyl cyclase activity and cAMP response to β-agonist as compared to non-asthmatic HASMCs. Confocal microscopy showed significant accumulation of phosphorylated β_2_ARs in asthmatic HASMCs. Systematic analysis of desensitization components including GRKs and β-arrestin showed no appreciable differences between asthmatic and non-asthmatic HASMCs. However, asthmatic HASMC showed significant increase in PI3Kγ activity and was associated with reduction in PP2A activity. Since reduction in PP2A activity could alter receptor resensitization, endosomal fractions were isolated to assess the agonist ready β_2_ARs as a measure of resensitization. Despite significant accumulation of β_2_ARs in the endosomes of asthmatic HASMCs, endosomal β_2_ARs cannot robustly activate adenylyl cyclase. Furthermore, endosomes from asthmatic HASMCs are associated with significant increase in PI3Kγ and reduced PP2A activity that inhibits β_2_AR resensitization. Our study shows that resensitization, a process considered to be a homeostasis maintaining passive process is inhibited in asthmatic HASMCs contributing to β_2_AR dysfunction which may underlie asthma pathophysiology and loss in asthma control.

## Introduction

β-adrenergic receptor (βAR) is a proto-typical member of a large family of seven transmembrane cell surface receptors termed G protein-coupled receptors (GPCR) [1,2] [3]. βAR consists of β_1_, β_2_ and β_3_ subtypes, of which β_2_AR is widely distributed in the respiratory tract and the most well studied in asthma [4–8]. β_2_AR-agonist (β_2_-agonist) binding evokes coupling of β_2_ARs to G-protein releasing Gs and Gβγ subunits. Gs-G protein activates adenylyl cyclase (AC) generating cAMP which in turn activates protein kinase A (PKA) that phosphorylates downstream targets mediating relaxation [9,10]. Dissociated Gβγ subunits recruit G-protein coupled receptor kinase 2 (GRK2) that phosphorylates β_2_ARs resulting in β-arrestin binding [11–13] desensitizing the receptors. β-arrestin targets β_2_ARs to undergo internalization but dissociates from β_2_AR complex prior to internalization [14]. β_2_ARs are resensitized by dephosphorylation through protein phosphatase 2A (PP2A) in the early endosomes before recycling to the plasma membrane as agonist ready receptors [15].

Although β_2_AR desensitization has been comprehensively studied, less is known about resensitization. Dephosphorylation of β_2_ARs by PP2A is a pre-requisite step in resensitization [2,16] and considered to be a passive homeostasis maintaining process. Contrary to this belief, our recent studies have shown that β_2_AR resensitization is tightly governed by regulation of PP2A [15,17]. PP2A is a serine-threonine phosphatase containing catalytic, scaffolding and regulatory subunits [18,19] which can be regulated by endogenously occurring inhibitor proteins called the inhibitor of PP2A, I1- and I2-PP2A [19,20]. We have shown that phosphoinositide 3-kinase γ (PI3Kγ) phosphorylates I2PP2A resulting in enhanced I2PP2A binding to PP2A inhibiting PP2A activity [17]. Inhibition of PP2A activity at the β_2_AR complex induces loss in resensitization due to incapability of PP2A in dephosphorylating receptors. In this context, whether altered resensitization contributes to β_2_AR dysfunction in pathology remains unknown.

β_2_AR dysfunction occurs in various pathological conditions [1,9] including asthma [21]. β_2_-agonist is commonly used for acute rescue of asthma as a bronchodilator [1,22] which mediates relaxation of airway smooth muscle (ASM) via the cAMP-PKA pathway [12]. Despite β_2_-agonist mediating acute relief in airway obstruction, chronic usage of β_2_-agonist evokes tachyphylaxis and a rebound in airway hyper-responsiveness [23]. In addition, the short and long- acting β_2_ agonists interact with β_2_ receptors differentially altering the relaxation and duration of bronchodilation in asthmatic patients. Moreover, genetic polymorphisms of β_2_ have been reported to alter the response of the β_2_-AR receptors to β_2_-agonist [8]. Dataset analysis from multiple clinical trials show that around 70% of asthma patients on β_2_-agonist lose the β_2_-agonist-induced broncho-protection [24,25] and yet, the underlying causes remain unknown. We postulate that β_2_AR dysfunction may contribute to the asthma diathesis and the alterations in desensitization and/or resensitization may underlie the asthma pathophysiology. While the mechanisms of β_2_AR desensitization are well characterized [1], less is known about β_2_AR resensitization in general and nothing in human airway smooth muscle cells (HASMCs). To address whether changes in desensitization/resensitization underlies β_2_AR dysfunction in asthma, we characterized β_2_AR function in HASMCs derived from non-asthmatic and fatal asthmatic subjects to provide insights into pathways altered in human airways.

## Methods

### Human airway smooth muscle cells (HASMCs) cultures

Primary HASMCs were isolated from the lungs of de-identified donors with fatal asthma and non-asthma, phenotyped and were used in their 3^rd^— 5^th^ passages for the experiments as previously described [26]. Although information concerning the cause of death, gender, race and age of the donor is available including medical history describing no communicable disease, there are no unique identifiers that can link the subject’s identification to the tissue sample. Cells were received at their first passage from Dr. Panettieri’s lab (non-asthma cell lines N051912/1, N090712/1, N 101412/1, N010912/1, N061212/1, N082112/1, N012412/1 & N120511/1; asthma cells lines AS010513/1, AS011813/1, AS110112/1, AS110412/1, AS 110612/1, AS091511/1, AS101411/1 & AS113011/1). The cells were expanded and serum starved for 12 hours prior to treatments.

### Limitations and advantages of using primary human airway smooth muscles for the study

In the current studies, we have used primary human airway smooth muscle cells derived from patient lungs. A caveat and a limitation of primary culture studies is the selective amplification of sturdy cells in the isolated population from the lungs. However, this limitation is outweighed by advantages like a) that the cells are directly derived from the asthmatic lungs providing a platform to probe for alterations in the pathways underlying asthma pathology and b) that these may be the most receptive cells that may respond to treatment in pathology. Therefore, pathways identified in these cells will have significant translational impact.

### Ethics statement

Although our studies have utilized primary human airway smooth muscle cells, these have been derived from anonymous patient donors. The adult human tissue is provided by the National Disease Research Interchange (NDRI) and the International Institute for the Advancement of Medicine (IIAM) according to the procedures approved by the University of Pennsylvania Committee on Studies Involving Human Beings. As such, the human tissue is exempt from requiring IRB approval as the use of this tissue is not considered human studies by the University of Pennsylvania Committee on Studies Involving Human Beings.

### Purification of plasma membrane and early endosomes

Purification of plasma membrane and endosomes were performed as described previously [17] [27]. Briefly, cells were homogenized in ice-cold lysis buffer containing 5mM Tris-HCl (pH 7.5), 5 mM EDTA, 1 mM PMSF, and 2 μg/mL Leupeptin & Aprotinin respectively. Cell debris/nuclei were removed by centrifugation at 1000 X *g* for 5 minutes at 4°C. Supernatant was transferred to a new tube and subjected to centrifugation at 37, 000 X *g* for 30 minutes at 4°C. The pellet containing plasma membrane was re-suspended in 75 mM Tris-HCl (pH 7.5), 2 mM EDTA, and 12.5 mM MgCl_2_ while, the supernatant underwent one more round of centrifugation at 200, 000 X *g* for 1 hour at 4°C. The pellet containing the endosomes was recovered by re-suspension in 75 mM Tris-HCl (pH 7.5), 2 mM EDTA, and 12.5 mM MgCl_2_.

### βAR density, adenylyl cyclase (AC) activity and cAMP

βAR density was determined by incubating 20 μg of the membranes (plasma membranes or endosomes) with saturating concentrations of [^125^I]-cyanopindolol (250 pmol/L) alone or along with 40 μM Alprenolol for non-specific binding as described previously [28,29]. AC assays were carried out by incubating 20 μg of membranes (isolated plasma membranes or endosomes) at 37°C for 15 minutes with labeled α^[32]^P-ATP as previously described [30,31]. ISO was used instead of albuterol in the plasma membranes/endosomal resensitization experiments because ISO is a full agonist which allows for higher G-protein coupling resulting in measurable levels of in vitro cAMP generation especially in the endosomal fractions. The cAMP content was determined in the cytosol using catch point cAMP kit (Molecular Devices; Sunnyvale, CA) as per manufacturer’s instruction [29].

### Phosphatase assay

Phosphatase activity was measured using the serine/threonine phosphatase kit (Upstate Biotechnology) as previously described [29]. Briefly, PP2A was immunoprecipitated and the immunoprecipitates were re-suspended in the phosphate-free assay buffer. The immunoprecipitated PP2A was incubated with phospho-serine/threonine hexa-peptide substrate specific for PP2A to assess for the loss in phosphorylation. The reaction was terminated by adding acidic malachite green solution and absorbance was measured at 630 nm in a plate reader (SpectraMax Plus 384, Molecular Devices).

### Immunoprecipitation and Immunoblotting

Immunoblotting and detection of proteins were carried out as previously described [29]. Cells were harvested in NP40 lysis buffer containing 20 mM Tris pH 7.4, 137 mM NaCl, 1% NP-40, 1 mM PMSF, 20% Glycerol, 10 mM NaF, 1 mM Sodium Orthovanadate, 2μg /ml Leupeptin and Aprotinin. The lysates were cleared by centrifugation at 12000 X *g* for 15 min at 4°C and the supernatants used for immunoprecipitation. Similarly, plasma membrane and endosomal fractions were re-suspended in the NP40 lysis buffer and processed for immunoprecipitation. PP2A was immunoprecipitated using anti-PP2A antibody (1:100) (Upstate Biotechnology), PI3Kα, β, δ and γ were immunoprecipitated using anti-PI3Kα (1:100), anti-PI3Kβ (1:100), anti-PI3Kδ (1:100) and anti-PI3Kγ (1:100) antibodies (Santa Cruz Biotechnology). The antibodies were incubated overnight at 4°C with lysates and protein A/G agarose beads and the immunoprecipitates were subjected to assays or western immunoblotting. Immuno-precipitates were washed and resolved by SDS-PAGE and transferred onto PVDF membranes (BIO-RAD) for western immunoblotting analysis. The membranes were blocked with 5% milk or 5% BSA and incubated with antibodies recognizing phospho-S-355/356-β_2_AR (Santa Cruz Biotechnology) at 1:1000 dilution, GRK, 2, 3, 5 and 6 (Santa Cruz Biotechnology) at 1:1000 dilution, β-Arrestin (BD biosciences, San Jose, California, 1: 300), PI3Kα and γ at 1:1000 (Santa Cruz Biotechnology) or I2-PP2A (Santa Cruz Biotechnology) at 1:1000 dilution. Following primary antibody incubation, appropriate secondary antibody (1:3000) was used and detection was carried out using enhanced chemiluminescence. Quantitative densitometric analysis was carried out using the NIH image J software.

### Confocal microscopy

Confocal microscopy was performed as previously described [29]. Non-asthmatic and asthmatic HASMCs were plated on poly L-Lysine treated cover slips. The cover slips containing non-asthmatic and asthmatic HASMCs without any treatment were fixed in 4% paraformaldehyde for 30 minutes, permeabilized with 0.3% Triton-X 100 and incubated in 1% BSA in 1 X PBS for 1 hour. After washing three times in 1 X PBS, the cover slips were incubated with anti-phospho serine (355/356) S-355/356-β_2_AR antibody (1:500; SantaCruz Biotechnology) in 1% BSA in 1 X PBS for 1hour. Following three washes with 1 X PBS, the cells were incubated with goat anti-rabbit IgG conjugated with AlexaFlour 488 (1:500; Molecular probes, Eugene, OR) for 1hour. The cells were washed in 1 X PBS, fixed with Vectashield (Vector Laboratories, CA) and β_2_AR phosphorylation visualized by sequential line excitation at 488 for green, with the correct emission filters. In each experiment, 100 to 120 positive cells were analyzed and the experiments were repeated with four different non-asthmatic and asthmatic HASMCs.

### Lipid kinase assay

Lipid kinase assays were performed on immunoprecipitated proteins as previously described [32]. PI3Kα, β, δ and γ were immunoprecipitated from cell lysates, plasma membranes or endosomes of non-asthmatic or asthmatic HASMCs and the immunoprecipitates were washed with buffers as described previously [33] and resuspended in 50 μl of reaction assay buffer containing 10 mM Tris-Cl (pH 7.4), 150 mM NaCl, 5 mM EDTA, 100 μM sodium-orthovanadate, 100 μM MgCl2, and 10 μl of 2 mg/ml phosphatidylinositol (PtdIns) (20 μg) sonicated in TE buffer (10 mM Tris-Cl, pH 7.4 and 1 mM EDTA). Reactions were started by adding 10 μl of 440 μM ATP and 10 μCi ^[32P]^Pγ-ATP and incubated at 23°C for 10 minutes with continuous agitation on a thermal mixer. The reaction was stopped with 20 μl 6N HCl and lipids were extracted by adding 160 μl of chloroform:methanol (1:1). After centrifugation, 30 μl of the organic phase was spotted onto 200 μm silica-coated TLC plates (Selecto-flexible; Fischer Scientific, Pittsburgh, PA) that were pre-coated with 1% potassium oxalate. The lipids were resolved using thin layer chromatography with 2N glacial acetic acid:1-propanol (1:1.87). The plates were dried, exposed, and lipid phosphorylation visualized using autoradiography.

### Statistics

Data are expressed as mean ± SEM. Statistical comparisons were performed using an unpaired Student’s *t*-test for two samples comparison and for multiple comparisons, two way analysis of variance (ANOVA) was used. Post-hoc analysis was performed with a Scheffe test. For analysis, a value of *P<0*.*05* was considered significant.

## Results

### Asthmatic HASMCs manifest β_2_AR dysfunction

**Fig 1:**
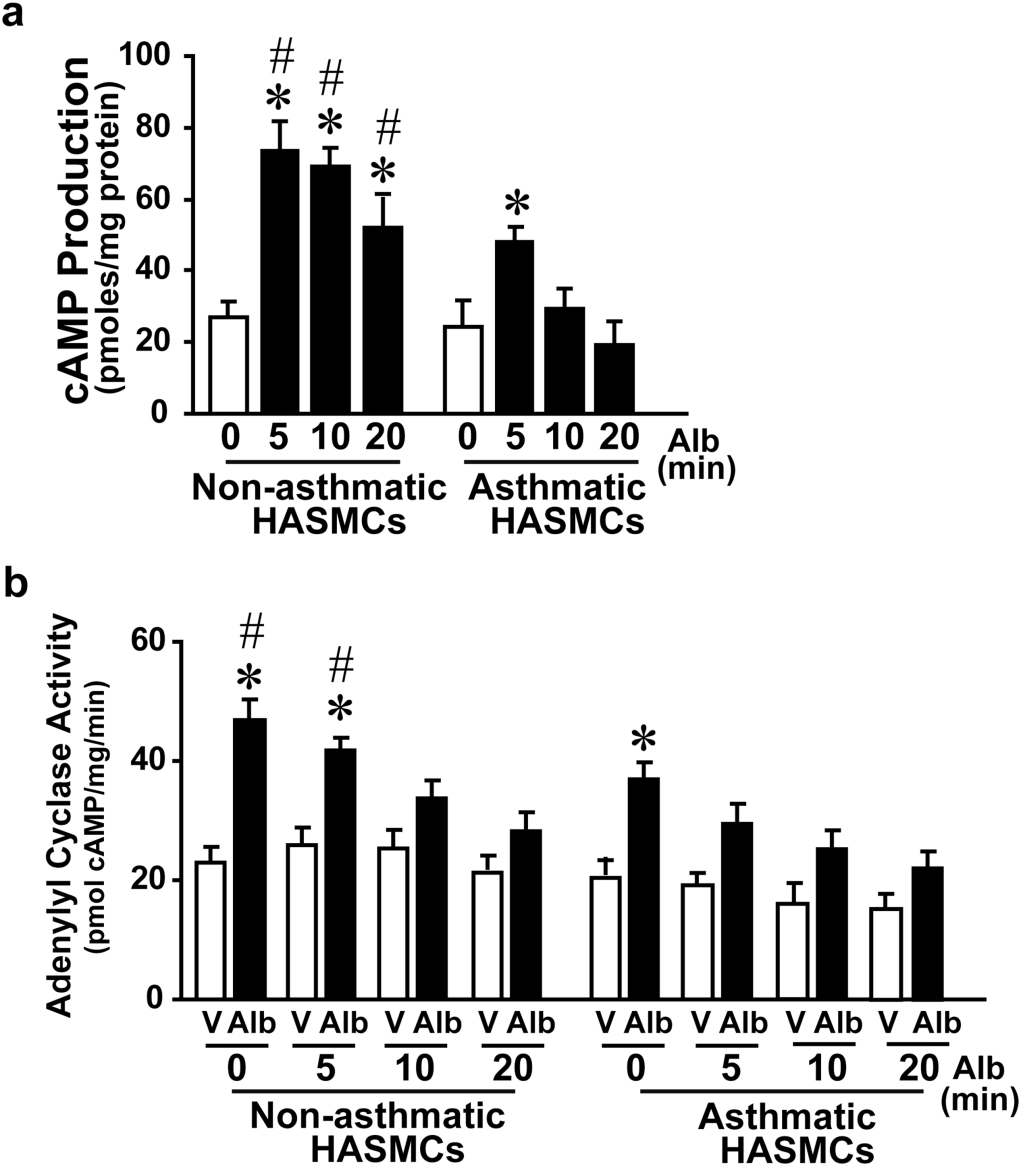
βAR function in primary human airway smooth muscle cells (HASMCs) from lungs of non-asthma (Non- asthmatic ASM) and asthma (Asthmatic ASM) patients. **a**, Non- asthmatic ASM and asthmatic ASM cells were stimulated with β-agonist albuterol (0, 5, 10 & 20 minutes (min)). The cell were lysed and assessed for the ability to generate cAMP. *p<0.005 vs. non-asthmatic ASM or asthmatic ASM 0 min (untreated), #p<0.05 vs. asthmatic ASM 5, 10, 20 min, (n = 6 per group, 6 non-asthmatic ASM and 6 asthmatic ASM). **b**, Plasma membranes were isolated from non-asthmatic ASM and asthmatic ASM cells following pre-treatment of cells with albuterol for 0, 5, 10, and 20 min. The cell-free membranes were stimulated with albuterol to measure adenylyl cyclase activity by providing radioactive ^32[P]^γ-ATP and measuring cAMP generation. *p<0.01 vs. respective *in vitro* vehicle stimulation, #p<0.05 vs. ASM *in vitro* albuterol stimulated 0 and 5 min, (n = 6/group). V, Vehicle; Alb, Albuterol (β-agonist).

To test whether asthmatic ASM is characterized by β_2_AR dysfunction, non-asthmatic and asthmatic HASMCs were challenged with albuterol (β-agonist) acutely (0, 5, 10 and 20 minutes) and cAMP generation assessed as a measure of βAR function. Sustained cAMP generation in asthmatic HASMCs in response to albuterol was not as robust as non-asthmatic HASMCs (Fig 1a) suggesting βAR dysfunction. To test whether βAR dysfunction in asthmatic ASM is due to loss in G-protein coupling, non-asthmatic and asthmatic HASMCs were treated with albuterol (0, 5, 10 or 20 minutes). Following treatment, plasma membranes were isolated and re-challenged with albuterol to determine adenylyl cyclase (AC) activation. Significant albuterol-stimulated cell free AC activity was observed in the plasma membranes from non-asthmatic HASMCs without albuterol pre-treatment followed by progressive reduction in AC activity with increasing albuterol pre-treatment (5, 10 or 20 minutes) (Fig 1b). In contrast, AC activity was markedly reduced in asthmatic HASMCs following albuterol (5, 10 or 20 minutes) compared to non-asthmatic HASMCs. These observations show that βARs in asthmatic HASMCs are pre-disposed to dysfunction due to their reduced G-protein coupling capability following agonist activation which is exacerbated even with shorter duration of β-agonist treatment.

### Augmented β_2_AR phosphorylation and loss of cell surface receptors in asthmatic HASMCs

**Fig 2:**
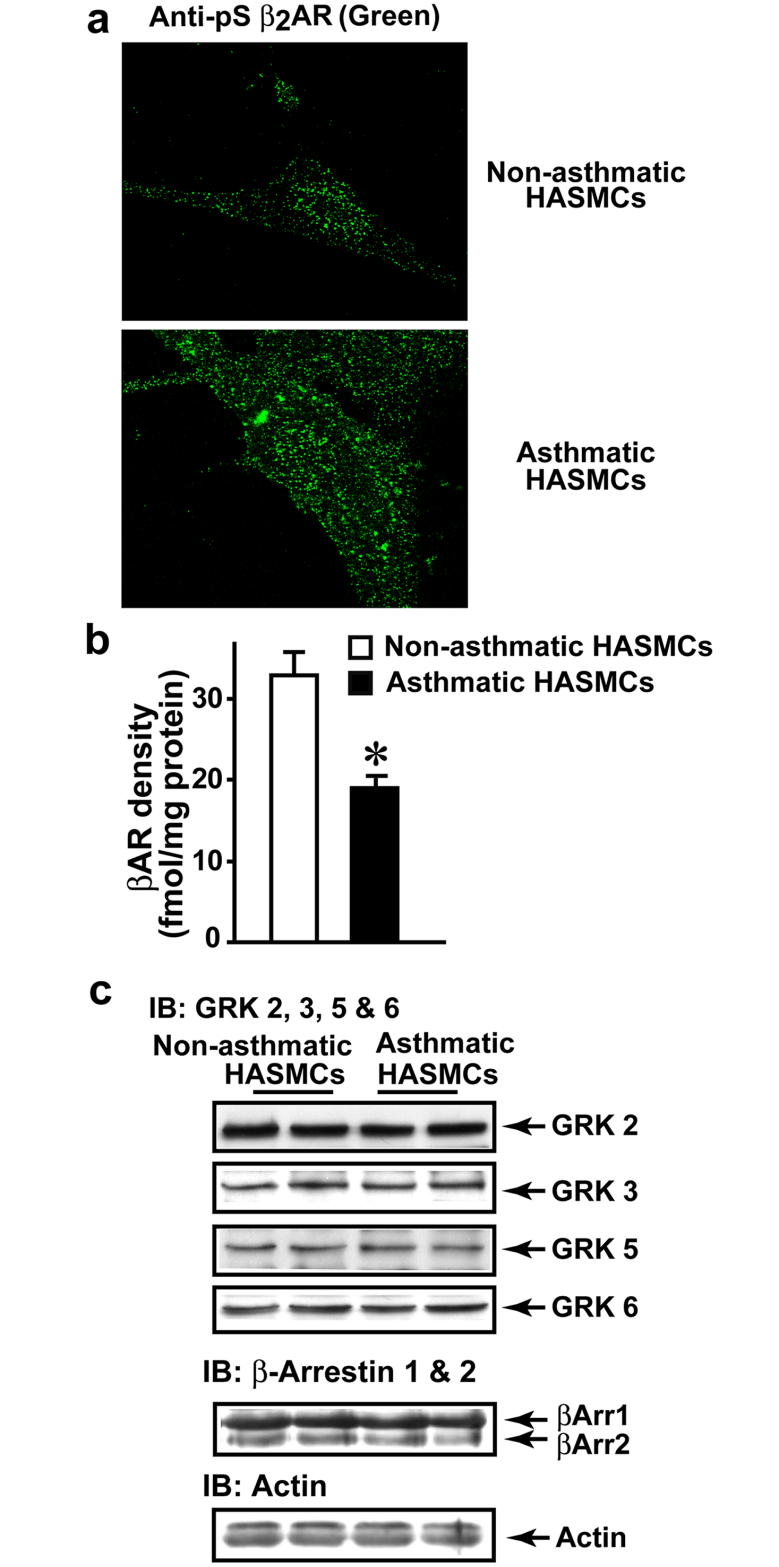
Assessment of βAR desensitization in non-asthmatic ASM and asthmatic ASM cells. **a**, To determine whether βAR are differentially phosphorylated in non-asthmatic ASM and asthmatic ASM cells, the cells were plated on cover slips and β_2_AR phosphorylation was visualized by confocal microscopy using anti-phospho-S-355/356 β_2_AR antibody (green) (Scale-100 μM) (n = 5/group). **b**, Plasma membranes isolated from non-asthmatic ASM and asthmatic ASM cells were subjected to ^[125]^I-CYP (cyanopindalol) βAR binding at saturation concentration of 250 pmol. *p<0.005 vs. non-asthmatic ASM, (n = 7/group). **c**, To investigate changes in desensitization βAR components lysates (100 μg) from non-asthmatic ASM and asthmatic ASM cells were immunoblotted for ubiquitously expressed GRKS, GRK2, 3, 5 or 6. The blots were stripped for each probing. Furthermore, the blots were immunoblotted for β-arrestin 1 and 2. Actin was blotted as loading control (n = 7/group).

Since βAR dysfunction is observed in asthmatic HASMCs, state of β_2_AR phosphorylation was assessed as an underlying cause for reduced receptor function. Non-asthmatic and asthmatic HASMCs were plated on cover slips and baseline β_2_AR phosphorylation determined by confocal microscopy using anti-phospho-β_2_AR antibody. Marked β_2_AR phosphorylation (green) was visualized in asthmatic HASMCs compared to non-asthmatic (Fig 2a) indicating that increased phosphorylation in part, may underlie a βAR defect in asthma HASM. Since significant β_2_AR phosphorylation was observed in asthmatic HASMCs, we assessed βAR density by radio-ligand binding. Plasma membranes from asthmatic HASMCs had significantly lower βAR density compared to non-asthmatic HASMCs (Fig 2b) consistent with the paradigm that receptor phosphorylation is a trigger for β_2_AR internalization. These data suggests higher endosomal accumulation of phosphorylated β_2_ARs in asthmatic HASMCs. Thus, in addition to loss in G-protein-AC coupling, lower cell surface receptor density may also contribute towards reduced cAMP generation in the ensuing β_2_AR dysfunction (Fig 1).

### Molecules regulating β_2_AR desensitization remain unchanged in asthma HASMCs

Increased β_2_AR phosphorylation is classically associated with increased desensitization mediated by up-regulation of GRK2 [30,34]. To test whether increased β_2_AR phosphorylation in asthmatic HASMCs is associated with changes in molecules regulating desensitization, lysates from asthmatic and non-asthmatic HASMCs were immunoblotted for the ubiquitously expressed GRKs, GRK 2, 3, 5 and 6 [35,36]. Despite significant β_2_AR phosphorylation, no differences in expression of GKR 2, 3, 5 or 6 were observed between non-asthmatic and asthmatic HASMCs (Fig 2c). Additionally, no differences in β-arrestin expression was observed (Fig 2c) suggesting that desensitization mechanisms may not account for increased phosphorylation of β_2_ARs in asthmatic HASMCs.

### PI3K activation in asthmatic HASMCs

**Fig 3:**
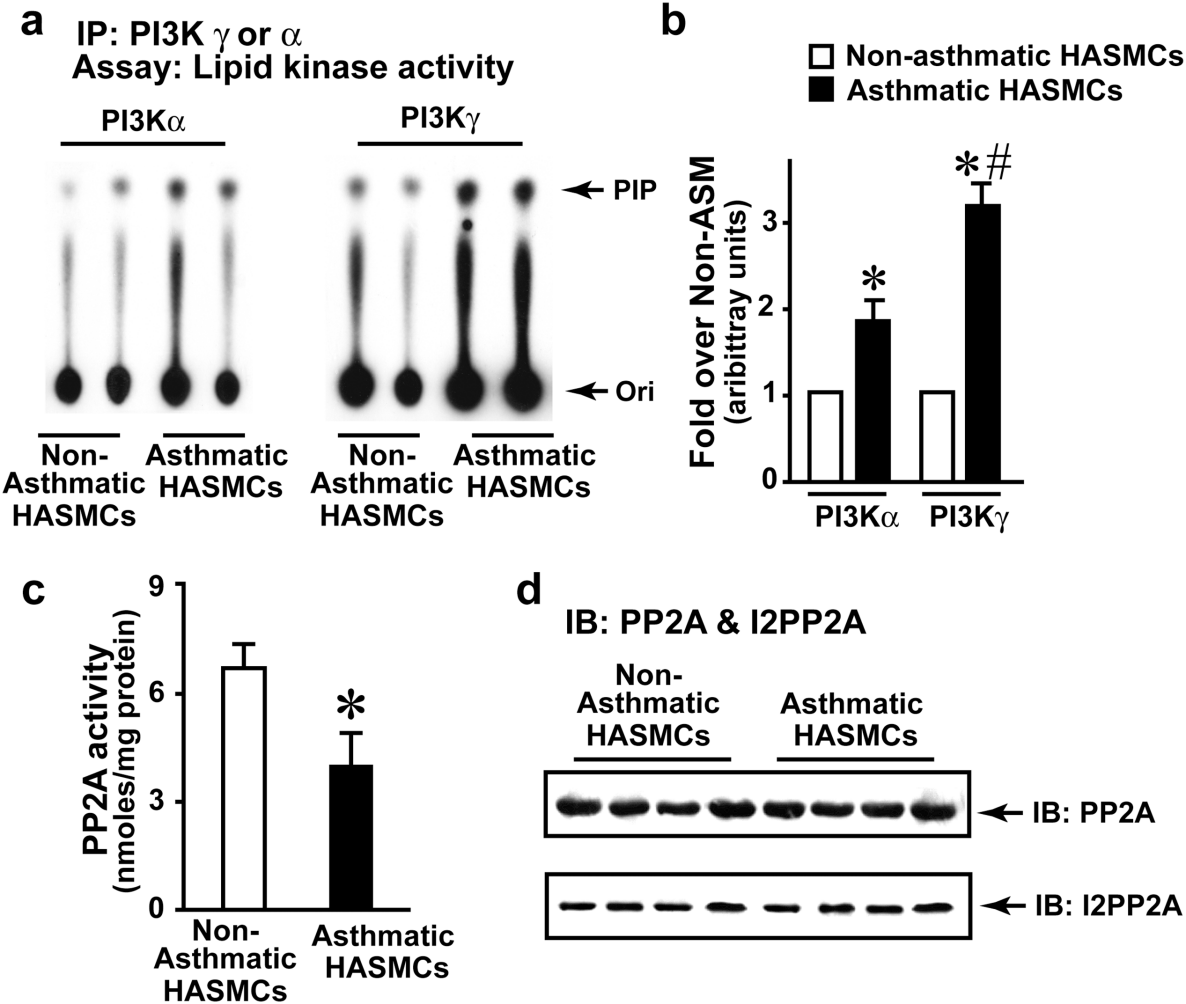
Investigate βAR resensitization components in non-asthmatic ASM and asthmatic ASM cells. **a**, PI3Kα or γ were immunoprecipitated from non-asthmatic ASM and asthmatic ASM cells (500 μg) and the immunoprecipitates were washed with lysis and kinase buffers. These immunoprecipitates were then subjected to *in vitro* lipid kinase assay by providing ^32[P]^γ-ATP to assess the phosphorylation of the Phosphatidylinositol (PI) as substrate. The reaction was then loaded on the TLC plate and resolved to identify formation of PIP. **b**, Densitometry of lipid kinase experiments (n = 7/group), *p<0.001 vs. non-asthmatic ASM, #p<0.05 vs. asthmatic ASM PI3Kα, (n = 7/group). **c**, To test whether PP2A activity is altered, PP2A was immunoprecipitated from (500 μg) of non-asthmatic ASM and asthmatic ASM cells using anti-PP2A antibodies and the immunoprecipitates were subjected to *in vitro* phosphatase assay with malachite green as a read for activity. *p<0.001 vs. non-asthmatic ASM, (n = 7/group). **d**, To investigate changes in βAR resensitization/dephosphorylation component, lysates (100 μg) from non-asthmatic ASM and asthmatic ASM cells were immunoblotted for PP2A expression. The blots were stripped and re-immunoblotted for inhibitor of PP2A, I2-PP2A (n = 7/group). Ori, Origin, PIP, Phosphatidylinositol-*bis*-phosphate.

Absence of alterations in desensitization molecules suggests that β_2_AR dysfunction in asthmatic HASMCs could be due to changes in resensitization which is regulated by PI3Kγ [29]. However, little is known about PI3K isoforms or their activity in HASMs. Therefore, activity of PI3Kα, β, δ and γ isoforms were determined by immunoprecipitating PI3Kα, β, δ and γ from non-asthmatic HASMCs and assayed for lipid kinase activity. Significant PI3Kα, δ and γ activity was observed compared to PI3Kβ suggesting that PI3Kα, δ and γ isoforms (S1a and S1c Fig) may play a critical role in HASM PI3K signaling. To test whether PI3Kα or γ is activated by albuterol treatment, non-asthmatic HASMCs were subjected to albuterol treatment and assessed for activation. Albuterol treatment activated PI3Kγ while minimal changes were observed with PI3Kα (S1b Fig). To determine whether PI3K activity is altered in asthma, PI3Kα, δ or γ were immunoprecipitated from asthmatic and non-asthmatic HASMCs. PI3Kα and γ activity was significantly higher in asthmatic HASMCs compared to non-asthmatic (Fig 3a and 3b) with no appreciable difference in PI3Kδ activity (S1c Fig). Interestingly, PI3Kγ was markedly higher in asthmatic HASMCs compared to PI3Kα (Fig 3a and 3b). As PI3Kγ is activated by Gβγ-subunits of G-proteins [37,38], we assessed whether muscuranic receptor agonists activate PI3Kγ. In contrast to albuterol, carbachol (a muscarinic receptor agonist) did not activate PI3Kγ (S1d Fig). However, carbachol still activated downstream extracellular regulated kinase (ERK) (assessed by phospho-ERK (pERK)) (S1e Fig) suggesting dissociation of PI3Kγ activation from muscuranic receptor-mediated downstream signaling.

### Asthmatic HASMCs are characterized by significant loss in PP2A activity

As phosphatase activity of PP2A mediates dephosphorylation and resensitization of β_2_ARs, PP2A was investigated to test whether changes in PP2A activity may underlie β_2_AR dysfunction in asthmatic HASMCs. Significant reduction in PP2A activity was observed in the PP2A immunoprecipitates from asthmatic HASMCs compared to non-asthmatic HASMCs suggesting that alterations in dephosphorylation mechanisms may underlie β_2_AR dysfunction (Fig 3c). However, immunoblotting showed no appreciable differences in expression of PP2A in asthmatic versus non-asthmatic HASMCs (Fig 3d, upper panel) indicating mechanisms of regulating PP2A function as a cause for loss in activity. Since the regulatory B subunit of PP2A does not play a role in activity but only in its localization [18], we assessed for the known endogenous regulator of the PP2A activity, inhibitor of PP2A—I2PP2A [20]. Interestingly, we observed no discernible differences in I2-PP2A expression between the asthmatic and non-asthmatic HASMCs (Fig 3d, lower panel) suggesting that changes in PP2A activity are not dependent on expression but might involve PI3Kγ-mediated phosphorylation of I2PP2A, that may then bind to PP2A inhibiting PP2A activity [29].

### Reduced β_2_AR resensitization in the endosomes of asthma HASMCs

**Fig 4:**
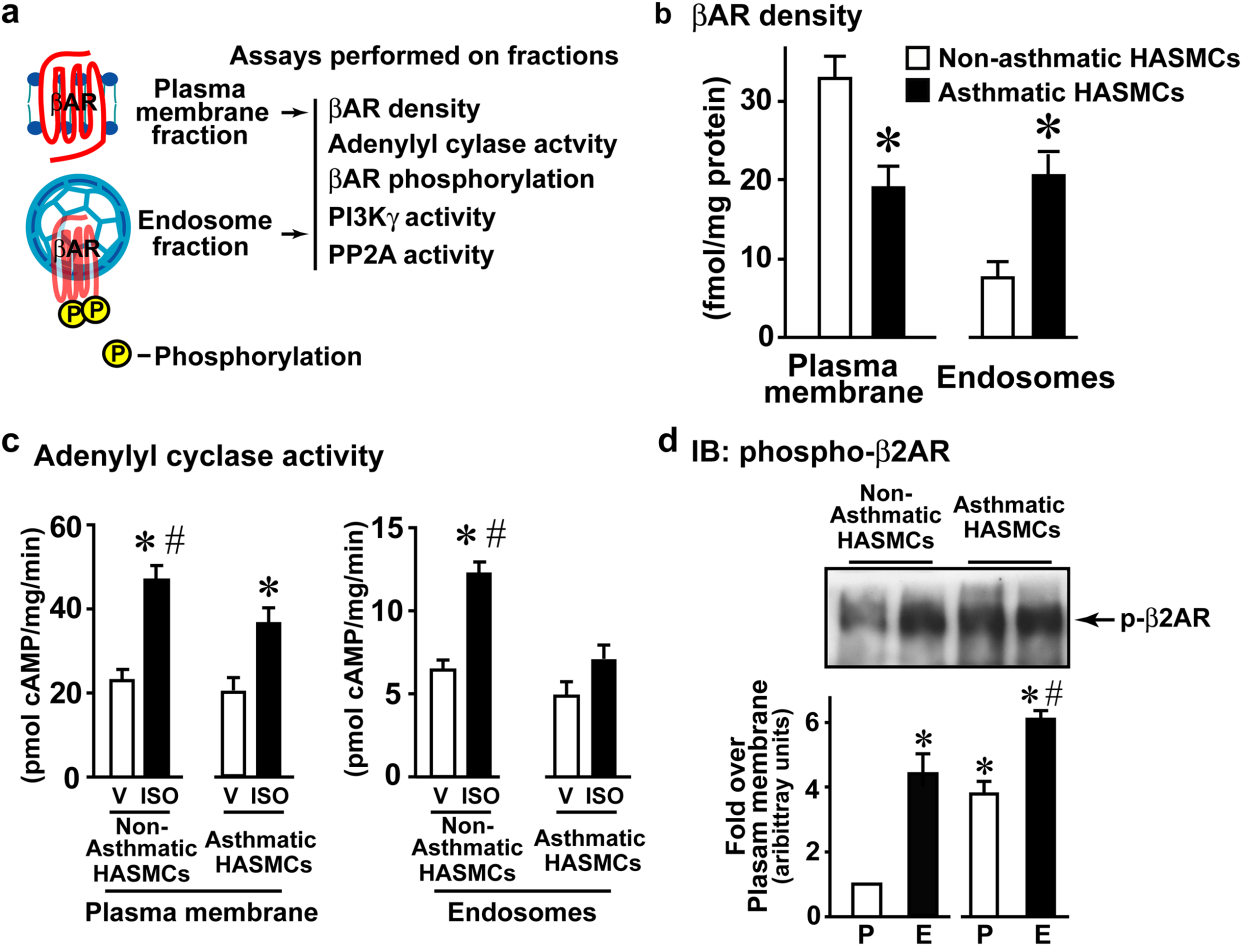
Measure of βAR resensitization in non-asthmatic ASM and asthmatic ASM cells (βAR function, density and distribution). **a**, To obtain a measure of resensitization that mediates βAR dephosphorylation leading to generation of agonist ready receptors in the endosomes, endosomes were isolated from non-asthmatic ASM and asthmatic ASM cells, and subjected to various assays. **b**, Plasma membranes and endosomes isolated from non-asthmatic ASM and asthmatic ASM cells were subjected to ^[125]^I-CYP (cyanopindalol) βAR binding at saturation concentration of 250 pmol. *p<0.005 vs. Non-ASM, (n = 4/group). **c**, Plasma membranes and endosomes isolated from non-asthmatic ASM and asthmatic ASM cells were subjected to cell-free membrane associated β-agonist (isoproterenol, ISO)-stimulated adenylyl cyclase activity. *p<0.01 vs. respective cell-free vehicle (V) stimulation, #p<0.05 vs. asthmatic ASM ISO, (n = 4/group). **d**, Upper panel, plasma membranes and endosomes isolated from non-asthmatic ASM and asthmatic ASM cells (100 μg) were immunoblotted using anti-phospho-S-355/356 β_2_AR antibody. Lower panel, densitometry for phospho- β_2_AR (n = 4/group). *p<0.01 vs. plasma membrane (P) non-asthmatic ASM, #p<0.05 vs. endosomes (E) non-asthmatic ASM. V, Vehicle, ISO, isoproterenol (β-agonist), P, Plasma membrane, E, Endosome.

β_2_AR dephosphorylation and resensitization occurs in the endosomes [2] and a recent study in HEK 293 cells showed that the dephosphorylated β_2_AR can be activated in the recycling endosomes [29]. To harness this observation within the context of resensitization, we established methods to biochemically measure β_2_AR resensitization in the HASMCs by isolating plasma membranes and endosomes, and subjecting them to β_2_AR functional coupling assays (Fig 4a). Consistent with our previous observation (Fig 2b), plasma membrane fraction from asthmatic HASMCs had significantly lower βAR density (Fig 4b). In contrast, endosomal fraction from asthmatic HASMCs had significantly higher βAR density compared to the non-asthmatics HASMCs (Fig 4b) suggesting that asthmatic HASMCs are characterized by endosomal accumulation of βARs. Importantly, total βAR density (sum of plasma membrane and endosomal fraction) was similar in asthmatic and non-asthmatic HASMCs (Supplementary Fig 1f) suggesting that receptor numbers may not be a critical determinant of asthma phenotype.

**Fig 5:**
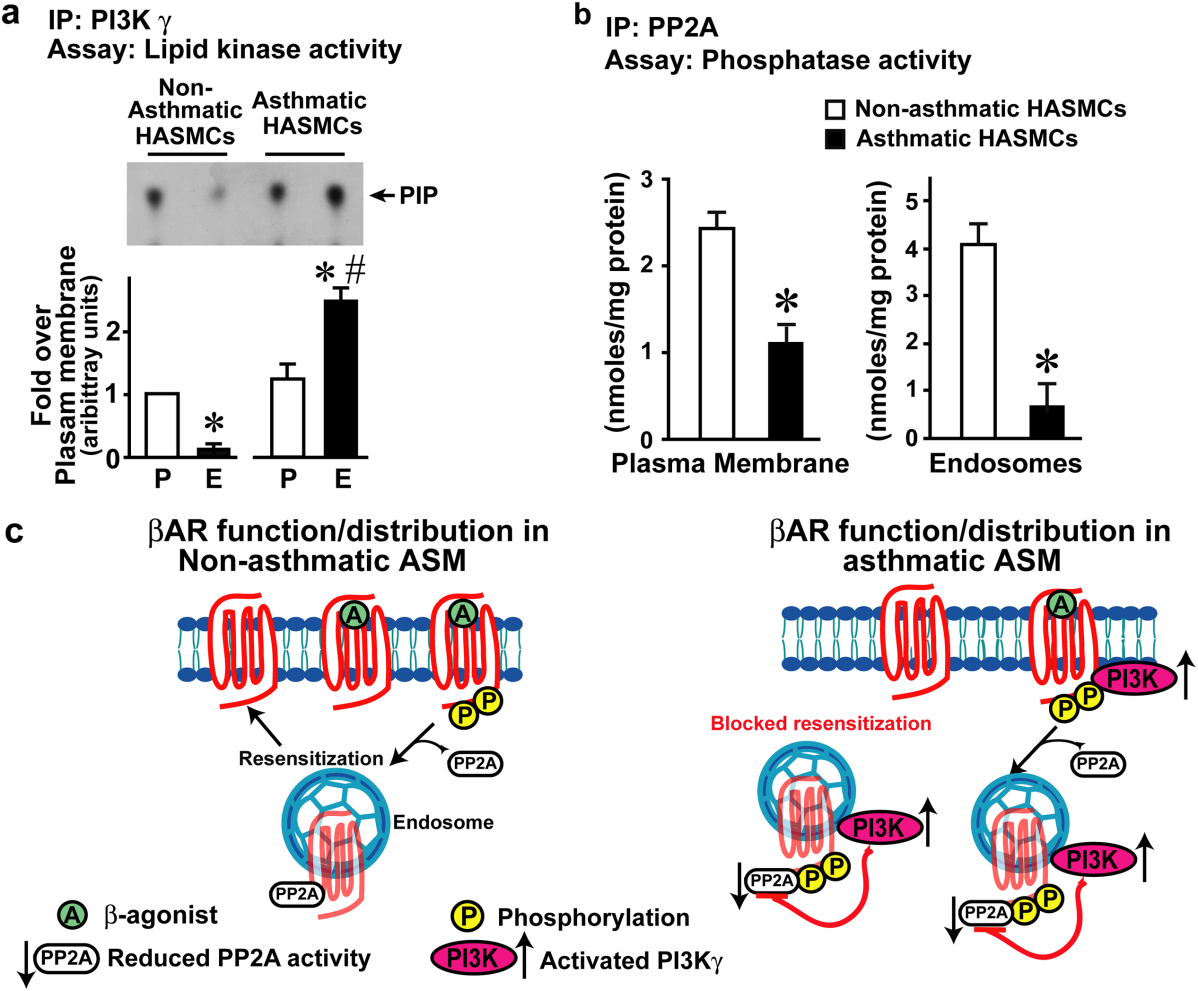
Measure of βAR resensitization in Non-ASM and ASM HASMCs (measure of resensitization components PI3Kγ and PP2A activity). **a**, Upper panel, PI3Kγ was immunoprecipitated from plasma membrane and endosomes isolated from non-asthmatic ASM and asthmatic ASM cells (120 μg) and the washed immunoprecipitates were subjected to *in vitro* lipid kinase assays. Lower panel, densitometry for PI3Kγ (n = 4/group). *p<0.01 vs. plasma membrane (P) non-asthmatic ASM, #p<0.0001 vs. endosomes (E) non-asthmatic ASM. **b**, PP2A was immunoprecipitated from (100 μg) of non-asthmatic ASM and asthmatic ASM cells using anti-PP2A antibodies and the immunoprecipitates were subjected to *in vitro* phosphatase assay with malachite green as a read for activity, (n = 4/group). *p<0.001 vs. non-asthmatic ASM,). **c**, Illustration depicting the loss of resensitization as a underlying cause for βAR dysfunction leading to paradoxical loss in β-agonist response. Left Panel (non-asthmatic ASM conditions), βAR activation by β-agonist leads to desensitization by phosphorylation and resensitization by dephosphorylation via PP2A resulting in normal βARs recycling. Right Panel (asthmatic ASM conditions), desensitization is well understood but, little is known of resensitization mechanisms. Since we have shown that βAR resensitization is regulated by PI3Kγ-PP2A axis, assessment in the endosomes shows marked increase in PI3Kγ activity associated with loss in PP2A activity. Based on this observation, we propose that asthma HASMCs are characterized by loss in βAR resensitization wherein, increased PI3Kγ inhibits PP2A activity blocking receptor resensitization resulting in accumulation of βARs in the endosomes.

To determine the functional capability of these βARs to couple G-protein and adenylyl cyclase (AC), plasma membrane and endosomal fraction were assessed for isoproterenol (ISO)-stimulated cell-free AC activity. ISO was used instead of albuterol because ISO is a full agonist which may allow for higher G-protein coupling resulting in measurable levels of in vitro cAMP generation especially in the endosomal fractions. Corresponding to our previous data (Fig 1b), plasma membrane AC activity was less robust in asthmatic HASMCs compared to non-asthmatic (Fig 4c). Interestingly, significant adenylyl cyclase activity was observed in the endosomes from non-asthmatic HASMCs (Fig 4c) which was markedly abolished in asthmatic HASMCs (Fig 4c) showing that endosomal βARs in the asthmatic HASMCs are unable to couple G-proteins. Since state of βAR phosphorylation is a critical determinant of β-agonist response, β_2_AR phosphorylation was assessed in the plasma membranes and endosomes. Significant β_2_AR phosphorylation was observed in the endosomal fraction (E) from the non-asthmatic HASMCs compared to minor β_2_AR phosphorylation at the plasma membranes (P) (Fig 4d). Remarkably, significant β_2_AR phosphorylation was observed both at the plasma membrane (P) as well as in the endosome (E) from the asthmatic HASMCs (Fig 4d). These data suggest that increased receptor phosphorylation underlies the reduced β_2_AR responses to agonist challenge. Therefore, we assessed whether inhibition of resensitization could be a cause for accumulation of phosphorylated β_2_ARs by assaying for PI3Kγ and PP2A activity. PI3Kγ activity was observed in the plasma membrane (P) fraction from both asthmatic and non-asthmatic HASMCs (Fig 5a). In contrast, endosomal (E) fraction from asthmatic HASMCs had significantly higher PI3Kγ activity compared to non-asthmatic HASMCs (Fig 5a) suggesting that increased endosomal PI3Kγ activity may attenuate βAR resensitization/dephosphorylation by inhibiting PP2A. To test whether elevated PI3Kγ is associated with reduction of PP2A activity, PP2A activity was assayed from plasma membranes and endosomes. Significant loss in PP2A activity was observed on the plasma membranes as well as endosomes in the asthmatic HASMCs (Fig 5b) compared to non-asthmatic HASMCs (Fig 5b) suggesting that reduction in phosphatase activity and loss in resensitization may underlie βAR dysfunction in asthma HASM.

## Discussion

β-agonist remains the most commonly used reversal therapy in acute asthma [9,10] and yet, accumulating evidence shows loss in β_2_AR-mediated broncho-protection. β_2_AR signaling in ASM is key for maintaining the bronchomotor tone and relaxation [23]. In our current study, we identified that asthmatic HASMCs have significant β_2_AR dysfunction compared to non-asthmatic HASMCs and may underlie the airway hyper-responsive phenotype in asthma. Critically, our studies show that the β_2_AR dysfunction in asthmatic HASMCs is not due to enhanced desensitization but reduced resensitization. Reduced resensitization is characterized by diminished dephosphorylation of β_2_ARs due to decreased endosomal PP2A activity that underlies the accumulation of the phosphorylated β_2_ARs in asthmatic HASMCs. Consistent with increased phosphorylation, endosomal βARs are unable to couple G-proteins to activate AC in HASMCs from fatal asthma patients. In contrast, endosomal βARs from non-asthmatic HASMCs couple to G-protein and activate AC indicating robust dephosphorylation of receptors in the endosomes thus, increasing the availability of agonist-ready βARs. Corresponding to accumulation of phosphorylated βARs in endosomes, asthmatic HASMCs have reduced endosomal PP2A activity and increased PI3Kγ activity that may contribute to βAR dysfunction by inhibiting resensitization (Fig 5c).

Airway tone and responsiveness is thought to be regulated by contraction and relaxation mediated by muscarinic and β_2_ARs respectively [9,39]. In ASM cells, the muscarinic receptor couples to inhibitory G-protein-Gi reducing cAMP levels evoking contraction which is reversed by β_2_AR coupling to stimulatory G-protein-Gs resulting in cAMP generation and relaxation [12]. Despite this intricate balance, alterations in these pathways are known to underlie the airway hyper-responsive phenotype in asthma [10]. Studies show that enhanced contractile tone of the airways is robustly inhibited by muscarinic receptor antagonist [40]. Conversely, β_2_-agonist i.e., long-acting β-agonist, has proved effective in ameliorating airway hyper-responsiveness to acute asthma symptoms [9,41]. However, chronic β_2_-agonist use evokes loss of asthma control as nearly 70% of severe asthma patients fail to broncho-protect with β-agonist [24,25,42], but it is not known whether the loss is due to altered desensitization or resensitization. In this context, recent study in mouse airway smooth muscle has shown the presence of both β_1_AR as well as β_2_ARs [47]. Nevertheless, overwhelming number of studies including those in the HASMCs has shown that β_2_AR is the key player [49, 50]. Thus, understanding the mechanisms that regulate β_2_AR function is critical and our studies show that in asthmatic HASMCs resensitization pathways may be altered contributing to receptor dysfunction. In addition to the alterations in resensitization mechanisms, chronic β-agonist treatment in asthma patients could lead to alterations in the β_2_AR signaling pathways that may also contribute to loss in asthma control. Interestingly, studies have shown elevated phosphodiesterase 4 (PDE4) in asthmatic HASMCs which could also accelerate the catalysis of cAMP [45] and contribute to loss in β_2_AR response. Similarly, PI3Kγ activation in the asthmatic HASMCs (Fig 3a & 3b) could also be a part of the altered β_2_AR downstream response inhibiting β_2_AR signaling as a feed-back mechanism. Thus, loss of β_2_ARs from cells surface, elevated PDE activity [45] and increased PI3Kγ activity in asthmatic HASMCs together could contribute towards significant reduction in robust β-agonist response and may underlie the exacerbation observed with chronic use of β-agonist.

Studies in various cells including non-asthmatic HASMCs have shown that β-agonist induces acute β_2_AR desensitization upon GRK-mediated phosphorylation associated with β-arrestin recruitment [1, 12] that reduces G-protein coupling [23]. Though β_2_AR dysfunction is known to occur in variety of diseases [16], it has been attributed to desensitization [36] mechanisms due to associated upregulation of GRK2-mediated phosphorylation [34, 48]. However, our studies show that β_2_AR dysfunction in asthma may be due to changes in resensitization mechanisms owing to reduced dephosphorylation rather than enhanced phosphorylation. This suggests that resensitization as a process could be as important as desensitization in determining β_2_AR functional outcome. Consistent with our observation of altered resensitization, asthmatic HASMCs have no appreciable upregulation of GRK 2, 3, 5 or 6 (the most ubiquitously expressed GRKs [43]). Furthermore, β-arrestin is also not altered in asthmatic HASMCs compared to the non-asthmatic suggesting that proximal molecules initiating β_2_AR desensitization are not altered. This observation may in part, explain the immediate beneficial effects observed with β-agonist in acute asthma events [10] while chronic use results in subsequent loss of this benefit [9]. This suggests a delicate dynamic balance which could be altered due to changes in downstream β_2_AR pathways including PI3Kγ activation in response to chronic β-agonist treatment.

Despite little change in proximal desensitizing molecules, asthmatic HASMCs are characterized by accumulation of β_2_ARs in the endosomes, an observation consistent with previous studies in asthma lungs [7]. Thus, chronic agonist treatment in asthma may promote progressive reduction in available β_2_ARs for agonist activation with endosomal accumulation, a characteristic biochemical phenotype in asthmatic HASMCs. Correspondingly, acute agonist stimulation of the asthmatic HASMCs results in markedly lower cAMP generation even over short time periods (20 minutes). The decrease in cAMP generation and subsequent reduction in adenylyl cyclase activity to acute agonist stimulation in asthmatic HASMCs is reflected by the reduced number of β_2_ARs available for agonist activation. Such a phenomenon explains the rebound in airway hyper-responsiveness following chronic β_2_-agonist treatment [44] suggesting that passive accumulation of β_2_ARs in the endosomes may underlie the asthma phenotype.

Loss in β-agonist response associated with minimal changes in desensitization machinery and significant accumulation of phosphorylated β_2_ARs in the endosomes of asthmatic HASMCs points to impaired resensitization in asthma etiology. In contrast to well the understood desensitization [17], little is known about resensitization. Resensitization has long been considered a passive homeostasis maintaining process wherein, phosphorylated β_2_ARs are dephosphorylated in the endosomes and recycled back as naïve receptors ready for agonist activation [1]. Due to this traditionally held paradigm, the idea that changes in the pace of dephosphorylation could alter the rate at which resensitization occurs has never been interrogated. Our study shows that reduction/inhibition of resensitization in asthmatic HASMCs promotes accumulation of phosphorylated β_2_ARs in the endosomes suggesting that resensitization could be dysregulated/impaired in pathology. Cellular studies have shown that PP2A that dephosphorylates βARs is negatively regulated by PI3Kγ [29]. Correspondingly our studies in asthmatic HASMCs show significant activation of PI3Kγ associated with marked reduction in PP2A activity. These observations show that the mechanisms regulating resensitization pathways are markedly impaired and may contribute to the asthma phenotype. In this context, impaired PP2A activity leads to a shift in the balance towards accumulation of phosphorylated β_2_ARs in the endosomes without altering the rate of desensitization. Consistent with this idea, we observe accumulation of βARs in the endosomes of asthmatic HASMCs as phosphorylated βARs undergo dephosphorylation in the endosomes [29].

Resensitization *per se* till now has always been described within the frame work of desensitization. Therefore, to test directly whether resensitization is altered, we employed the classical receptor activation assays but on endosomal fractions in light of the recent study in HEK 293 cells showing that β_2_ARs in recycling endosomes can be activated by agonist [2,46]. We sought to take advantage of this observation by performing cell free βAR stimulated adenylyl cyclase activation assays as a measure of resensitization in the endosomes. Since resensitization (dephosphorylation of receptors) is known to occur in the endosomes, significant PP2A activity in the endosomes of non-asthmatic HASMCs would lead to concomitant βAR dephosphorylation resulting in generation of agonist ready receptors. In contrast, inhibition of resensitization would lead to reduced PP2A activity and impaired dephosphorylation resulting in accumulation of phosphorylated βARs in the endosomes. Consistently, endosomal fractions from asthmatic HASMCs have significantly lower agonist ready receptors as measured by adenylyl cyclase activity compared to non-asthmatic HASMCs. Additionally, the endosomes from asthmatic HASMCs had marked reduction in PP2A activity associated with increased PI3Kγ mechanistically supporting the idea that elevated PI3Kγ inhibits PP2A in the endosomes impairing resensitization. Impaired resensitization consequently leads to accumulation of βARs in the endosomes as observed in asthmatic HASMCs. Thus, our findings show that resensitization of βAR is altered in the human airway smooth muscle cells in severe asthma accounting for the rebound in airway hyper-responsiveness and ironic loss in response to β-agonist treatment.

These studies suggest a paradigm shift that β_2_AR resensitization considered passive and homeostasis maintaining, could be dysregulated to actively evoke disease pathogenesis. These findings are very important as it suggests that resensitization which has been overlooked up until now could be an integral contributing factor in pathology. Our data shows that changes in resensitization results in βAR dysfunction, that in part could evoke airway hyper-responsiveness. Further studies on the molecular mechanisms regulating β2AR resensitization will provide key insights into developing therapeutic strategies to target βAR resensitization in asthma [41]. Finally, our study provides an understanding on why acute β-agonist treatment is beneficial in asthma while, chronic treatment paradoxically does not provide the expected relief in asthma.

## Supporting Information

S1 Fig **a**, PI3Kα, β or γ were immunoprecipitated from non-asthmatic HASMCs (500 μg) and the immunoprecipitates were washed and subjected to vitro lipid kinase assay to measure the generation of PIP. The reaction was then loaded on the TLC plate and resolved to determine formation of PIP. **b**, Non-asthmatic HASMCs were stimulated with albuterol for 0, 5, 10 and 20 minutes (min). Plasma membranes were isolated and PI3Kα or γ was immunoprecipitated (150 μg) and the immunoprecipitates were subjected to *in vitro* lipid kinase assay to measure PIP generation. **c**, PI3Kδ was immunoprecipitated from Non-asthmatic and asthmatic HASMCs (500 μg) and the immunoprecipitates were washed with lysis and kinase buffers before being subjected to vitro lipid kinase assay. **d**, Non-asthmatic HASMCs were stimulated with albuterol (β-agonist) or carbachol (muscuranic receptor agonist) for 0, 5, 10 and 20 minutes (min). Plasma membranes were isolated, PI3Kγ was immunoprecipitated (150 μg) and the immunoprecipitates were subjected to *in vitro* lipid kinase assay to measure generation of PIP with β-agonist or muscuranic agonist. Since carbachol, a muscuranic receptor agonist (G-protein coupled receptor agonist) did not activate PI3Kγ, the lysates were blotted for phospho-ERK (**e**) to demonstrate that carbachol activates downstream signals in non-asthmatic HASMCs. **f**, Total βAR density shown as a sum additive of βAR density from plasma membrane and endosomal fraction from non-asthmatic and asthmatic HASMCs (n = 4/group).
(TIF)
