# Supplementary figures and images for: Defective Resensitization in Human Airway Smooth Muscle Cells Evokes β-Adrenergic Receptor Dysfunction in Severe Asthma
Source: PLoS One. 2015 May 29;10(5):e0125803. doi: 10.1371/journal.pone.0125803 (PMC4449172; doi:10.1371/journal.pone.0125803)

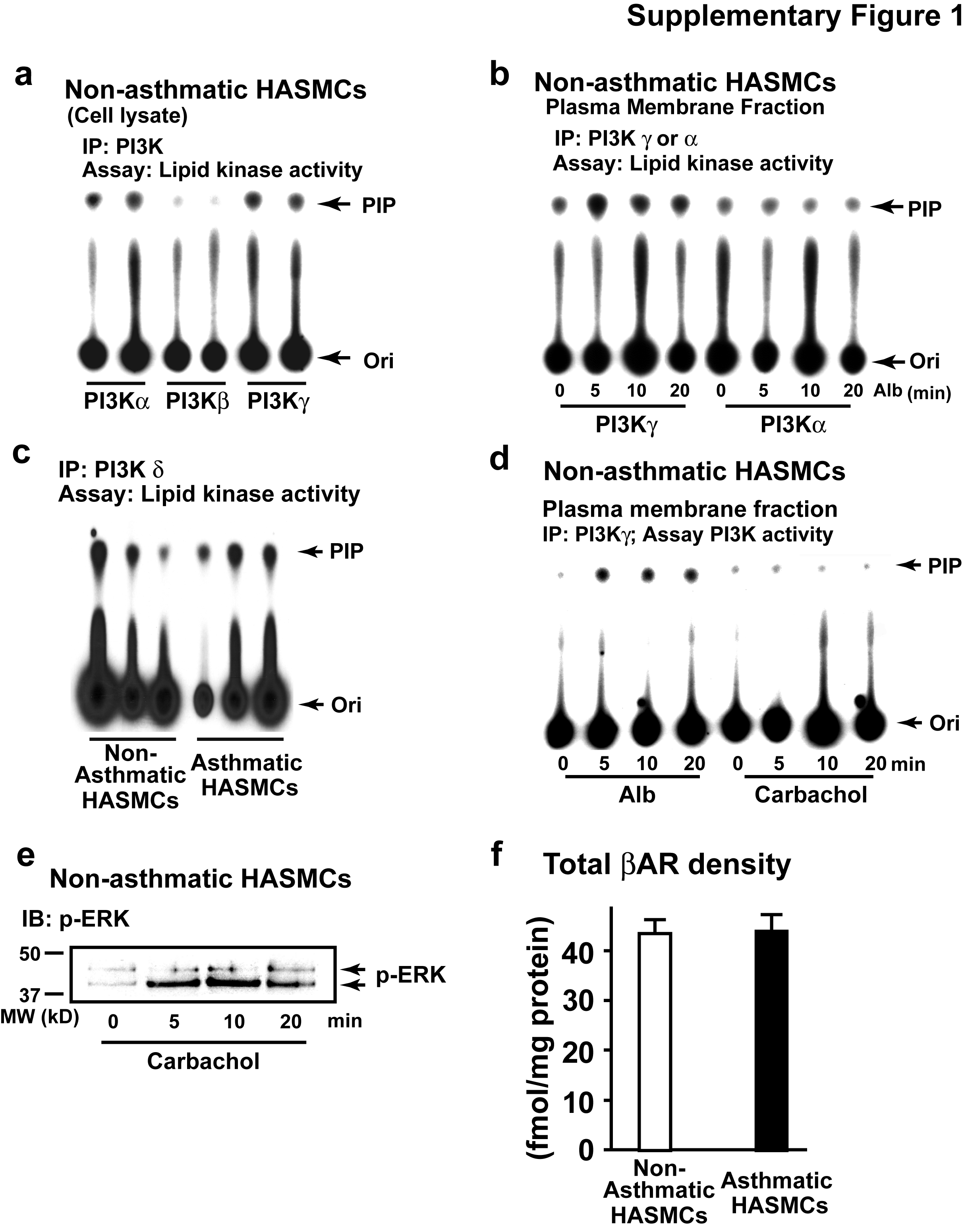

Supplement: S1 Fig — a, PI3Kα, β or γ were immunoprecipitated from non-asthmatic HASMCs (500 μg) and the immunoprecipitates were washed and subjected to vitro lipid kinase assay to measure the generation of PIP. The reaction was then loaded on the TLC plate and resolved to determine formation of PIP. b, Non-asthmatic HASMCs were stimulated with albuterol for 0, 5, 10 and 20 minutes (min). Plasma membranes were isolated and PI3Kα or γ was immunoprecipitated (150 μg) and the immunoprecipitates were subjected to in vitro lipid kinase assay to measure PIP generation. c, PI3Kδ was immunoprecipitated from Non-asthmatic and asthmatic HASMCs (500 μg) and the immunoprecipitates were washed with lysis and kinase buffers before being subjected to vitro lipid kinase assay. d, Non-asthmatic HASMCs were stimulated with albuterol (β-agonist) or carbachol (muscuranic receptor agonist) for 0, 5, 10 and 20 minutes (min). Plasma membranes were isolated, PI3Kγ was immunoprecipitated (150 μg) and the immunoprecipitates were subjected to in vitro lipid kinase assay to measure generation of PIP with β-agonist or muscuranic agonist. Since carbachol, a muscuranic receptor agonist (G-protein coupled receptor agonist) did not activate PI3Kγ, the lysates were blotted for phospho-ERK (e) to demonstrate that carbachol activates downstream signals in non-asthmatic HASMCs. f, Total βAR density shown as a sum additive of βAR density from plasma membrane and endosomal fraction from non-asthmatic and asthmatic HASMCs (n = 4/group). (TIF) [file pone.0125803.s001.tif]
